# Gene expression profiling in chicken heterophils with *Salmonella *enteritidis stimulation using a chicken 44 K Agilent microarray

**DOI:** 10.1186/1471-2164-9-526

**Published:** 2008-11-06

**Authors:** Hsin-I Chiang, Christina L Swaggerty, Michael H Kogut, Scot E Dowd, Xianyao Li, Igal Y Pevzner, Huaijun Zhou

**Affiliations:** 1 Department of Poultry Science, Texas A&M University, College Station, TX 77843, USA; 2 United States Department of Agriculture, Agricultural Research Service, Southern Plains Agricultural Research Center, College Station, TX 77845, USA; 3 Research and Testing Laboratories and Medical Biofilm Research Institute, Lubbock, TX 79407, USA; 4 Cobb-Vantress, Inc., Siloam Springs, AR 72761, USA

## Abstract

**Background:**

*Salmonella *enterica serovar Enteritidis (SE) is one of the most common food-borne pathogens that cause human salmonellosis and usually results from the consumption of contaminated poultry products. The mechanism of SE resistance in chickens remains largely unknown. Previously, heterophils isolated from broilers with different genetic backgrounds (SE-resistant [line A] and -susceptible [line B]) have been shown to be important in defending against SE infections. To dissect the interplay between heterophils and SE infection, we utilized large-scale gene expression profiling.

**Results:**

The results showed more differentially expressed genes were found between different lines than between infection (SE-treated) and non-infection (control) samples within line. However, the numbers of expressed immune-related genes between these two comparisons were dramatically different. More genes related to immune function were down-regulated in line B than line A. The analysis of the immune-related genes indicated that SE infection induced a stronger, up-regulated gene expression of line heterophils A than line B, and these genes include several components in the Toll-like receptor (TLR) signaling pathway, and genes involved in T-helper cell activation.

**Conclusion:**

We found: (1) A divergent expression pattern of immune-related genes between lines of different genetic backgrounds. The higher expression of immune-related genes might be more beneficial to enhance host immunity in the resistant line; (2) a similar TLR regulatory network might exist in both lines, where a possible MyD88-independent pathway may participate in the regulation of host innate immunity; (3) the genes exclusively differentially expressed in line A or line B with SE infection provided strong candidates for further investigating SE resistance and susceptibility. These findings have laid the foundation for future studies of TLR pathway regulation and cellular modulation of SE infection in chickens.

## Background

Salmonellosis in humans often results from consuming foods contaminated with *Salmonella*. The reported incidences of human infections by *Salmonella *have dramatically increased since 1980, and at present are approximately 1,400,000 cases every year in the United States, which indirectly caused a significant economical loss due to medical costs and loss of productivity (Economic Research Service, ). *Salmonella *enterica serovar Enteritidis (SE) is one of the most common *Salmonella *serotypes in many countries including the US, and is the main source of human salmonellosis through the consumption of contaminated poultry or shell eggs [1]. SE is a zoonotic pathogen and persists in the chicken cecum or ovaries without triggering clinical signs in the host. Salmonellosis in young chickens may cause high mortality as a result of severe diarrhea and dehydration, and include a greater risk of evolving into a carrier state [2-4].

Salmonella organisms can reach distal ileum and cecum in infected birds as the first place through oral route [5]. The outcome of an encounter with *Salmonella *is dependent on multiple factors including genetic background [6,7]. Although several studies have focused on the pathogenesis of SE in infected young chickens, the mechanism of SE resistance in healthy-carrier chickens remains unknown. Heterophils, the avian counterpart of mammalian neutrophils, are the most abundant leukocytes in the peripheral blood and are essential for initiating and modulating innate immunity [8]. Reducing the number of circulating heterophils significantly increases the susceptibility of young chickens to organ invasion by SE indicating a key role for peripheral blood heterophils in controlling SE infections in poultry [9]. It has also been reported that a large influx of heterophils is observed in the intestines of SE-infected chickens, indicating an increase in heterophils to the infection site contributes to defending against microbial infection [9,10]. Both studies of *in vivo *and *in vitro *SE-infected heterophils from different chicken lines also revealed that the up-regulated mRNA expression levels of *interleukin *(*IL*)*-1β*, *IL-6*, *IL-8 *(also known as *CXCLi2*), *IL-18*, and *anti-inflammatory cytokines transforming growth factor-β4 (TGF-β4) *might be responsible for determining overall immune competence [11,12]. We have extensively characterized the innate immune response of two parental broiler lines (designated lines A and B). To date, we have shown increased *in vitro *heterophil function [13]. corresponds with an increased *in vivo *resistance to organ invasion by SE [11]. In addition, we have shown increased mRNA expression levels of pro-inflammatory cytokines in heterophils isolated from the more resistant line compared to the susceptible line [11,12,14]. Collectively, the data indicate differences in heterophil function and innate responsiveness are under genetic control.

Heterophils play an important role in providing increased resistance against SE infections in poultry. The objective of the present study was to examine SE resistance by dissecting the interplay between heterophils and SE. Large-scale expression profiling technology including microarrays has been successfully used to achieve this goal [7,15-18]. Microarray technology provides a more comprehensive, unbiased knowledge of all gene networks including members of gene families, ligands, receptors, and transcription factors [19]. Additionally, microarray analysis allows for the discovery of new genes and/or pathways previously not known to be involved in a specific host-pathogen interaction. In the present study, a chicken genome Agilent microarray [20] was used to profile differential gene expression in heterophils from two genetically distinct parental broiler lines (SE-susceptible [line B] and -resistant [line A]) following *in vitro *stimulation with SE. The objectives of this study were to discover genes or gene networks associated with SE resistance and to examine the genetic effects on defending against SE infections in chicken heterophils.

## Results

### Identification of differentially expressed genes

**Figure 1:**
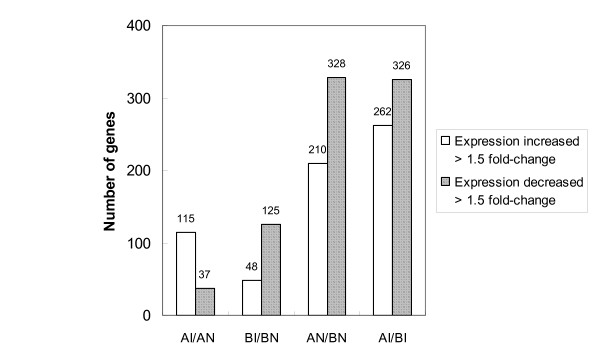
**Number of differentially expressed genes at four different comparisons**. Cut-off: Larger than 1.5 fold-change of increased or decreased expression with P-value smaller than 0.001.

The genome-wide expression profiling of each element (probe) was assigned to four different comparisons as AI/AN (line A infection vs. non-infection), BI/BN (line B infection vs. non-infection), AN/BN (non-infection line A vs. line B) and AI/BI (infection line A vs. line B). In this context, the word infection refers to *in vitro *stimulation with SE. In the microarray analysis, genes differentially expressed at *P *< 0.001 were considered statistically significant. The estimated false discovery rates at this level were controlled as 20%, 20%, 5%, and 5% in each comparison of AI/AN, BI/BN, AN/BN, and AI/BI, respectively. The fold-change range of gene expression differences between the groups were 21.61 to -4.21, 9.60 to -6.67, 29.16 to -6.91, and 33.30 to -4.57 in each comparison of AI/AN, BI/BN, AN/BN, and AI/BI, respectively (see Additional files 1, 2, 3, 4). The biological significance for each comparison was analyzed using the assigned cut-off expression ratio of 1.5 fold-change, and the direction of regulation. In general, more differentially expressed genes were found in the comparison between different lines (with 288 genes overlapped) than between infected and non-infected cells within line (with 51 genes overlapped) (Fig. 1). The regulation direction of these differentially expressed genes showed a dissimilar pattern in the comparisons of infected vs. non-infected cells between two lines. There were more genes up-regulated in the AI/AN (115 out of total 152 differentially expressed genes in AI/AN, and 48 out of total 173 differentially expressed genes in BI/BN). However, more genes were down-regulated in the BI/BN (37 out of total 152 differentially expressed genes in AI/AN, and 125 out of total 173 differentially expressed genes in BI/BN). A similar pattern of line A vs. line B comparisons was observed between infected and non-infected cells in terms of the number of up-regulated and down-regulated genes. In general, line B showed higher mRNA level of gene expression than line A in both infected and non-infected cells.

### Gene ontology analysis

**Figure 2:**
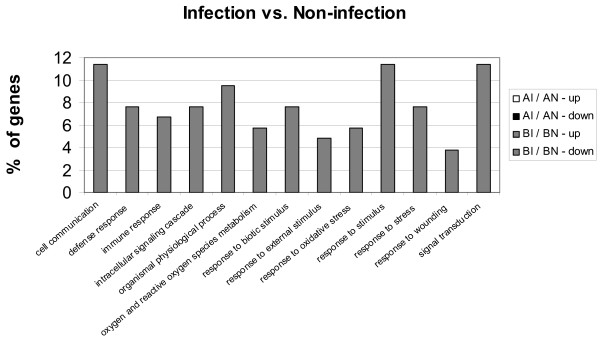
**Gene ontology (GO) annotation of differentially expressed genes (*P *< 0.001)**. A: Biological processes of up- or down-regulated genes between infection and non-infection treatments (I/N). B: Biological processes of up- or down-regulated genes between line A and line B heterophils (A/B). The percentage represents the enriched-intensity of each term. The percentage represents the enriched-intensity of each term.

The functional analysis was performed by identifying gene ontology (GO) terms (biological processes) of genes whose expression were significantly enriched among the pool of all differentially expressed genes. A Fisher-exact test was used to determine the enrichment of associated GO terms. Only significantly enriched (*P *< 0.05) GO terms are presented. In general, fewer significantly enriched GO terms were found in the comparison between infected and non-infected cells within line (Fig. 2A) than between genetic lines (Fig. 2B). In the comparisons between infected and non-infected cells, many functional terms were enriched in line B, while none of the functional terms were found significantly enriched in the same comparison for line A. For the down-regulated genes in the BI/BN comparison, many significantly enriched functional terms, including defense and immune response, and response to stress, were associated with the host defense system according to the GO term annotation [21]. For the line comparison, there were more enriched functional terms in the down-regulated genes than in the up-regulated genes. Interestingly, many enriched terms were overlapped between AN/BN and AI/BI with the similar abundance (%) of genes.

### Quantitative real-time PCR

Validation of the microarray data was performed using quantitative real-time PCR (qRT-PCR). This allowed us to: (1) confirm the microarray results across different comparisons, and (2) validate selected immune-related genes associated with *Salmonella *infection of heterophils. Eight significantly expressed genes were randomly selected for qRT-PCR confirmation (see Additional file 5). The samples used in qRT-PCR were not the same as in the microarray study but were obtained according to the same experimental design. The results showed that most of the genes selected for qRT-PCR (13 incidences of differential expression) analysis were consistent with the results obtained from the microarray (14 incidences of differential expression) (see Additional file 6). For the four inconsistencies, the fold-changes from three were very close to the microarray results.

### Immune-related genes

According to the information of the Gene Ontology Consortium's annotation [21], 426 immunologically-related genes (represented 542 probes in the array) were identified in this array. In order to study the host response to *Salmonella *infection and the role of genetic differences between the two lines, the list of immune-related genes were used to narrow down those previously identified differentially expressed genes (*P *< 0.001). Using the designated cut-off of 1.5 fold-change, 20 genes were found differentially expressed with SE infection (Table 1), where 12 genes were found in the comparison of line A and line B (Table 2). Several genes have duplicate probes in the array with consistent expression among comparisons. The number and regulation direction of immune-related genes showed a similar tendency to that of overall differentially expressed genes (Fig. 1B), in which there were more up-regulated genes in the AI/AN comparison, and more down-regulated genes in the BI/BN comparison. In the comparison between the genetic lines, fewer immune-related genes were identified with only a few genes that had differential expression overlapped between IA/IB and NA/NB. Since most immune-related genes showed positive fold-change ratio these data indicate that these genes have a stronger expression in line A than line B regardless of the SE infection.

**Table 1:** List of immune genes with differential expression (*P *< 0.001) between infection (I) and non-infection (N) treatment

**Accession No.**	**Gene Name**	**AI/AN^a ^fold-change**	**BI/BN^b ^fold-change**
AJ720630	Antizyme inhibitor 1 (AZIN1)	2.59	-
AJ851659	CD80 antigen	3.85	-
Y15006	Interleukin-1beta (IL1β)	3.40	-
AJ309540	Interleukin 6 (IL6)	11.39	8.96
AJ564201	Interleukin 12B (IL12B)	3.19	2.71
AJ720504	Toll-like receptor 7 (TLR7)	-4.21	-5.78
BX930367	PREDICTED: bactericidal/permeability-increasing protein	-2.01	-2.25
CR390308	Glioma Amplified Sequence 41	-1.90	-1.88
L34553	Chemokine (C-C) ligand 4 (CCL4)	7.30	3.59
M16199	Interleukin 8 (IL8)	7.40	4.03
M64990	Prostaglandin-endoperoxide synthase 2 (PTGS2)	3.14	3.19
Y14971	CXC chemokine K60 (K60)	8.93	4.12
AF176086	Similar to NUMB protein (NUMB)	-	-2.29
AF335427	Nuclear factor, interleukin 3 regulated	-	-1.97
AJ639839	Similar to immunoglobulin-like receptor B4	-	-5.55
AJ851768	Interleukin-1receptor-associated kinase 2 (IRAK2)	-	-2.05
AY621307	Beta-defensin 5 (GAL9)	-	-1.80
BU133261	Similar to Inhibitor of nuclear factor kappa-B kinase epsilon subunit (IKBKE/IKK-ε)	-	-5.18
BU265026	TIR domain containing adaptor inducing interferon-beta (TRIF/TICAM1)	-	-1.96
CR338732	TNF receptor-associated factor 7 (TRAF7)	-	-1.62

a,: The dash ("-") means that the expression differences were less than 1.5 fold. When the ratio (AI/AN) is smaller than 1, the ratio -(AN/AI) is given.

b,: The dash ("-") means that the expression differences were less than 1.5 fold. When the ratio (BI/BN) is smaller than 1, the ratio -(BN/BI) is given.

**Table 2:** List of immune genes with differential expression (*P *< 0.001) between chicken lineages A and B

**Accession No.**	**Gene Name**	**AI/BI^a ^fold-change**	**AN/BN^b ^fold-change**
AF176086	Similar to NUMB protein (NUMB)	2.18	-
AJ719428	TNF receptor-associated protein 1 (TRAP1)	-1.60	-
TC207578	Immunoglobulin-like receptor Ig1-37	1.93	-
U97157	Lunatic fringe homolog (LFNG)	1.89	-
CD735422	B-G V-region-like B-G antigen (B-G)	1.80	1.57
L34552	CC Chemokine (CCL1L2)	1.63	1.62
U20338	Interferon regulatory factor 7 (IRF7)	2.03	2.40
AF082329	Caspase 6, apoptosis-related cysteine peptidase (CASP6)	-	-2.68
AF320331	Interferon regulatory factor 4 (IRF4)	-	2.15
CV858509	TNF receptor-associated factor 2 (TRAF2)	-	1.66
L39766	Interferon regulatory factor 1 (IRF1)	-	2.01
Y12012	CD4 antigen (CD4)	-	1.71

a,: The dash ("-") means that the expression differences were less than 1.5 fold. When the ratio (AI/BI) is smaller than 1, the ratio -(BI/AI) is given.

b,: The dash ("-") means that the expression differences were less than 1.5 fold. When the ratio (AN/BN) is smaller than 1, the ratio -(BN/AN) is given.

### Utilization of the array

All microarray results from this study were deposited in NCBI's Gene Expression Omnibus (GEO) database [22]. The accession numbers are: Platform, GPL4993; Series, GSE9416; Samples, GSM239322, GSM239330, GSM239336, GSM239337, GSM239347, GSM239349-GSM239352, GSM239354-GSM23935456, GSM239358, GSM239370, GSM239372 and GSM23935473.

## Discussion

Evaluation of host responses to bacterial infections *in vitro *using microarray technology has become one of the major research areas in the study of functional genomics. This technology allows us to characterize the comprehensive host response(s) to complex pathogen stimuli under different experimental conditions. There has been a rapid increase in studies reporting the host response to *Salmonella *in chickens [2,23]. While most of the recent studies focus on profiling gene expression in immunologically-related tissues [15,16,18,24], the present study is the first to examine the response of a chicken innate immune leukocyte, the heterophil, to SE using microarray technology. Based on earlier findings, one hour post infection was selected as the point at which the peak cytokine mRNA expression levels were observed with *in vitro *SE stimulation in the two lines [14]. More time points following SE exposure to heterophils would provide interesting information regarding the kinetics of the host-pathogen interaction.

The FDR is used to control false positives in a declared significant gene in microarray studies. There are several factors affecting FDR: (1) the proportion of truly differentially expressed genes; (2) the distribution of true differences; (3) measurement variance; and (4) sample size [25]. Of these factors, the proportion of truly differentially expressed genes has the most significant effect on FDR [25]. The proportion of truly differentially expressed genes depends on the biological questions being addressed. Obviously, truly differentially expressed genes from the comparison between infection vs. non-infection and the comparison between line A and line B in the present study would be different. Therefore, using the same FDR for these two comparisons might miss many false negative genes if the proportion of truly differentially expressed genes in that comparison was small. In that case, using same cut-off of *P*-value is more appropriate than FDR although it is not optimal.

The microarray experimental design used in this study provided direct comparisons to identify differentially expressed genes due to SE infection (infection or non-infection) or genetic differences (line A or B). More differentially expressed genes were detected in the comparisons between the lines compared to that observed between SE infected and non-infected cells. These data indicate there may be an intrinsic genetic difference between line A and B chickens. Line A chickens have a stronger immune response against *in vivo *bacteria challenge than line B chickens [10], however, no further study has been conducted. In addition, fewer differentially expressed genes in the comparison between infected and non-infected cells within line may also be due to the limited variance contributed by host response associated with SE infection at one time point (1 h post infection). The direction of gene regulation revealed that line A (AI/AN) had less down-regulated genes, but more up-regulated genes compared to line B (BI/BN). Interestingly, similar patterns were observed on the expressional direction of immune-related genes (Table 1). Given that line A chickens are more resistant to SE than line B, it is possible that the enhanced SE-resistance is associated with a different host response in terms of both a higher number of up-regulated immune-related genes accompanied by fewer down-regulated genes.

### GO terms enrichment analysis

The analysis of enriched GO terms allowed us to discover significant categories that could be overlooked when evaluating individual genes. The enriched GO terms could aid in interpreting the dominant functions controlled by differentially expressed genes. Although the higher number of identified GO terms might be positively correlated to more differentially expressed genes identified, the regulation direction of genes showed a remarkable difference as most enriched GO terms were composed of down-regulated genes in all comparisons.

No specific functional term (biological process) was significantly enriched with SE infection in the comparison of AI/AN, while several functional terms associated with defense systems were found from down-regulated genes in the BI/BN comparison. The annotations of these terms suggest that line B may be more vulnerable to SE infections due to the suppressed functions on prevention or recovery from damages caused by infection. The results of the functional analysis further supported the results of expressional direction of immune-related genes, in which down-regulated genes (functional terms) with SE infection might be associated with the immuno-inefficiency observed in line B.

The comparison between different lines showed that most enriched functions had higher expression in line B than line A on both infected and non-infected cells. This suggested that these functions are not immune-related, and therefore the higher expression in line B might not benefit the host defense system. Although only three functions showed higher expression in line A, one of these functions named 'response to stress' might benefit line A by remaining normal under exposure to infections. Several functional terms related to metabolism (e.g. cellular metabolism, primary metabolism, and protein metabolism) were down-regulated in line A with SE infection. Although heterophils are well known as primary phagocytes in immune system, there might be a complicated interaction between the immune system and metabolism [26]. It is possible that the highly expressed functions in line B may serve as an advantage over other performances in different desired traits. More studies are needed in order to understand the effects of the line differences on other parameters.

### Analysis of immune-related genes

One of the key objectives of this study was to identify novel candidate genes associated with *Salmonella *resistance in chickens. The genetic variance contributing to the immune function only consist of a part of the whole genetic variance which is the overall genetic difference between line A and B. There were more differentially expressed genes in the comparisons between the two lines (538 and 588 genes in AN/BN and AI/BI, respectively) than observed in the comparisons between the infected and non-infected cells (152 and 173 genes in AI/BI and AN/BN, respectively). However, more immune-related genes were found in the comparisons between the infection and non-infection pairings (12 and 17 genes in AI/BI and AN/BN, respectively) than between the two lines (7 and 8 genes in AI/BI and AN/BN, respectively).

Numerous genes associated with immune function were found in both AI/AN and BI/BN pairing with a slightly higher fold-change in gene expression in AI/AN than BI/BN. These genes included the cytokines *IL-1β *and *IL-6*, and the chemokines *IL-8 *(*CXCLi2*), *CCL4 *and *K60 *(also known as *CXCLi1*). Cytokines and chemokines are essential for an effective innate immune response [27]. These data confirm and support earlier studies showing the higher expression of these signaling molecules in resistant line A are more important for their role in recruiting heterophils to the site of SE infection and pathogen clearance [28], and initiating the signaling cascades that promote a pro-inflammatory cytokine/chemokine response [16].

The effect of the MHC on determining immunity to Salmonella is described in the chicken [29,30]. There is a polymorphism (Lys148→Met148) in the MHC I α2 domain that is associated with bacterial load in the spleen following an SE challenge [31]. Interestingly, several genes involved in MHC II system were differentially expressed in the present study. These genes included *CD80, MHC II β chain *(accession no. U02881), *c-KIT *(*v-kit Hardy-Zuckerman 4 feline sarcoma viral oncogene*, accession no. D13225), *B-G *and *CD4*. *CD80*, *MHC II β *chain (*P *= 0.005) and *c-KIT *(*P *= 0.036) were up-regulated in the comparisons of AI/AN, and the expression of *B-G *and *CD4 *were higher in line A than line B on both infected and non-infected cells. Functionally, the CD80 antigen is a surface molecule that co-regulates with another surface molecule, CD86, to provide a co-stimulating signal for T- helper cell activation [32,33]. On the other hand, c-KIT is a stem cell factor receptor that is co-expressed with MHC II to sustain T-helper cell development [34]. Heterophils have never been shown to have a role in antigen presentation and subsequent development of an acquired immune response; however, these data are indicative that heterophils may actually have such a role and future experiments will be conducted to assess this possibility.

Defensins are small peptides composed of cysteine-rich cationic molecules with broad-spectrum antimicrobial activity against bacteria, fungi and enveloped viruses [35]. One of the families, namely β-defensins, is widely accepted as an important component for the hosts' immune system. It has been suggested that avian β-defensins play a significant role in the avian innate defense system since heterophils lack an oxidative killing mechanisms [36]. To date, 14 β-defensin genes, known as *gallinacin *(*GAL*) *1, 1A, 2–13*, are described in chickens [37-39]. In the present study, SE infection suppressed the gene expression of *β-defensin 5 *(*GAL 9*) on line B heterophils, while no significant effect was observed on line A heterophils. GAL 9 has stronger antimicrobial activity against *Salmonella *serovars than GAL 4 and 7 [40]. It is possible that repression of *GAL 9 *is related to the impaired SE-resistance in the susceptible line and lends itself as a potential candidate gene for selecting poultry with increased resistance against SE.

The TLR signaling pathway plays a critical role for elevating host immune responses by sensing pathogen-associated molecular patterns (PAMPs). Several genes associated with the TLR pathway have been reported to respond to *Salmonella *infection [12,41-44]. In the present study, two novel candidate adaptors, *IKK-ε *(*inhibitor of nuclear factor kappa-B kinase epsilon subunit*) and *TRIF *(*TIR domain containing adaptor inducing interferon-beta*), were found repressed exclusively in BI/BN. IKK-ε (also know as IKK epsilon or IKK-i) is an IKK homologue but not in components of IKK complex [45,46]. Although the underlying mechanisms remain elusive, it suggests that IKK-ε plays a role in the activation of IRF3 and NF-kB by involving TANK-binding kinase 1 (TBK1) [47-50]. TRIF is an adaptor of the MyD88-independent pathway that leads to interferon (IFN)-β production, and the downstream cascade of TRIF is directly regulated by the adaptors IKK-ε and TBK1 [51]. Interestingly, the co-repression of *TRIF *and *IKK-ε *in line B observed in the current study suggests an important role for MyD88-independent pathway in host defense. A few genes involved in the TLR pathway were not significant since a stringent cut-off *P*-value established, even though the *P*-values of these genes approached 0.001. While controlling false discovery rate is one of major objectives for microarray analysis, false negative might be an issue. In reality, it is possible that the lack of TLR-related expressed genes may in fact be one of the findings lost using stringent FDR criteria. These specific genes include receptors (*TLR4*, *TLR15*) and adaptors (*MD-2 like*, *MKK3*, *NFkB-1*) with *P*-values smaller than 0.05, and fold-changes larger than 1.5 in the comparisons between the infected and non-infected cells. Collectively, these findings support our assumption that the TLR pathway is, but probably not the only one, involved in altering host defense system to SE infection through a response of releasing signaling molecules differently as seen in cytokines and chemokines.

Most immune-related genes showed stronger expression in line A heterophils than in line B heterophils regardless of the SE infection. It is unclear if these genes are responsible for the stronger induction of immune response in the resistant line. Numb is an inhibitor of the notch signaling pathway that maintains normal cell-to-cell communication, cell fate specification and tissue regeneration [52,53]. In the current study, the expression of numb was suppressed with SE infection in line B, while there was a significant up-regulation in the AI/BI. Given that line B showed down-regulation in the function of cell communication with SE infection, it is possible that the suppressed numb in line B indirectly retards the host immune network through impaired cell communication.

## Conclusion

In summary, the results from this study demonstrate that higher expression of immune-related genes is more beneficial to enhance the host response against SE infection. The immune deficiency in the susceptible line is likely due to suppressed functions in recovering from cellular changes induced by SE infection. The genes exclusively differentially expressed in the AI/AN or BI/BN in the study has provided strong candidates for further investigation of disease resistance and susceptibility to SE infection in chickens, respectively. The identified immune-related genes also suggested a similar TLR regulatory network might exist in both lines, where a possible MyD88-independent pathway may participate in the regulation of host innate immunity in line B. Finally, the MHC II system might be important to initiate T-helper cell activation for the host defense.

To our knowledge, this is the first report to profile global gene expression in chicken heterophils with *in vitro Salmonella *infection. It is also expected that candidate genes discovered from this study along with the increasing information will add more genes to the chicken immune gene database. Although an *in vivo *study might be desirable to add additional insights regarding the interplay between heterophils and SE, the findings in this study have made an indispensable contribution to characterize the role of heterophils in the host immune system, and laid a solid foundation to further study the role of host genetics and resistance against *Salmonella*.

## Methods

### Experimental chickens

The two distinct parental meat-type broiler lines used in this study were obtained from a commercial company. To maintain confidentiality, the lines were designated A and B. At the day of hatch, chickens were placed in floor pens (8 feet) containing wood shavings, provided supplemental heat, water, and a balanced, un-medicated corn and soybean meal based chick starter diet *ad libitum*. The feed was calculated to contain 23% protein and 3200 kcal of metabolized energy/kg of diet, and all other nutrient rations met or exceeded the standards established by the National Research Council (1994). Animal experiments were conducted according to regulations established by the United States Department of Agriculture animal care and use committee (#2007003) and overseen by Dr. J. A. Byrd, attending veterinarian.

### Bacteria

A poultry isolate of SE (#97-11771) was obtained from the National Veterinary Services Laboratory (Ames, IA) and approved by the United States Department of Agriculture (USDA). SE was cultured in tryptic soy broth (Difco Laboratories, Becton Dickinson Co., Sparks, MD) overnight at 41°C. Stock SE (1 × 10^9 ^colony forming units [cfu]/ml) was prepared as previously described [13].

### Heterophil isolation

Heterophils were isolated from a pooled collection of peripheral blood from 6-day-old chickens (n = 100 for each line). Blood was collected on four separate occasions on age-matched, straight-run chickens. Following blood collection, heterophils were isolated as previously described [12]. Briefly, blood from chickens was collected in vacutainer tubes containing disodium ethylenediaminetetraacetic acid (EDTA) (BD vacutainer, Franklin Lakes, NJ) and mixed thoroughly. The blood and EDTA for each line was pooled and diluted 1:1 with RPMI 1640 media containing 1% methylcellulose and centrifuged at 40 *g *for 15 min at 4°C. The supernatant was transferred to a new conical tube and diluted with Ca^2+^- and Mg^2+^-free Hanks balanced salt solution (1:1), layered onto discontinuous Histopaque^® ^gradients (specific gravity 1.077 over 1.119) and centrifuged at 190 *g *for one h at 4°C. The histopaque layers were collected, washed with RPMI 1640 (1:1) and pelleted at 485 *g *for 15 min at 4°C. The cells were then re-suspended in fresh RPMI 1640, counted on a hemacytometer, and diluted to 1 × 10^7^/ml in RPMI. All tissue culture reagents and chemicals obtained from Sigma Chemical Company, St. Louis, MO, unless noted otherwise.

### Total RNA Isolation

Heterophils (1 × 10^7^) were treated with 300 μl RPMI or SE, for 1 h at 39°C on a rotary shaker at the ratio of multiplicity of infection = 20. Treated heterophils were pelleted, washed with RPMI (485 × *g *for 15 min at 4°C), the supernatant discarded, the cells re-suspended in lysis buffer (Qiagen RNeasy mini RNA extraction kit, Qiagen Inc., Valencia, CA), and frozen. The lysed cells were transferred to QIAshredder homogenizer columns and centrifuged for 2 min at ≥ 8000 × *g*. Total RNA was extracted from the homogenized lysate according to the manufacturer's instructions, eluted with 50 μl RNase-free water and stored at -80°C.

### Microarray experiment design

A dual color, balanced design was used to provide four different comparisons: AI/AN, BI/BN, AN/BN and AI/BI. Four biological replicates were conducted in each comparison and the dye balance was used throughout in order to prevent the dye-bias during the sample labeling.

### Labeling and hybridization

The integrity of total RNA samples was confirmed using Agilent Bioanalyzer 2100 Lab-on-chip system (Agilent Technologies, Palo Alto, CA, USA). Five hundred nanograms (ng) of total RNA were reverse-transcribed to cDNA during which a T7 sequence was introduced into cDNA. T7 RNA polymerase-driven RNA synthesis was used for the preparation and labeling of RNA with Cy3 (or Cy5) dye. The fluorescent cRNA probes were purified using Qiagen RNeasy Mini Kit (Qiagen Inc., Valencia, CA), and an equal amount (825 ng) of Cy3 and Cy5 labeled cRNA probes were hybridized on a 44 K chicken Agilent array (GEO accession: GSE9416). The hybridized slides were washed using a commercial kit package (Agilent Technologies, Palo Alto, CA, USA) and then scanned using Genepix 4100A scanner (Molecular Devices Corporation, Sunnyvale, CA) with the tolerance of saturation setting of 0.005%.

### Microarray data collection and analysis

For each channel, the median of the signal intensity and local background values were used. A Locally Weighted Linear Regression (LOWESS) normalization was applied to remove signal intensity-dependent dye bias for each array using R program. The normalized data was analyzed using commercial SAS 9.1.3 program (SAS Institute Inc. Cary, NC) with mixed model analysis. The mixed model used to identify significantly differentially expressed genes was:

Y_ijklm _= μ + T_i _+ L_j _+ D_k _+ S_l _+ T*L_ij _+ e_ijklm_

Where Y_ijklm _represents each normalized signal intensity; μ is an overall mean value; T_i _is the main effect of treatment (SE infection) i; L_j _is the main effect of chicken line j; D_k _is the main effect of dye k; S_l _is the random effect of slide l; T*L_ij _is the interaction between treatment and line; and e_ijklm _is a stochastic error (assumed to be normally distributed with mean 0 and variance σ^2^). An approximate F test on least-square means was used to estimate the significance of difference for each gene in each comparison where *P *< 0.001 was considered to be statistically different. The false discovery rate (Q value) was calculated for each *P*-value using R program according to the Storey and Tibshirani method [54].

### Quantitative real-time PCR

Total RNA (300 ng) from each sample (AI, AN, BI and BN) was used for cDNA synthesis with random hexamer primer of a Thermoscript RT-PCR system kit (Invitrogen, Carlsbad, CA) according to the manufacturer's manual. The cDNAs were quantified by qRT-PCR using ABI prism 7900HT system (Applied Biosystems, Foster, CA) with SYBR Green PCR Master Mix (Applied Biosystems, Foster, CA). The specific oligonucleotide primers were designed by PRIMER3 program [55]. The conditions of qRT-PCR amplification were: 1 cycle at 95°C for 10 min, 40 cycles at 95°C for 15 s and 59°C for 1 min. The chicken β-actin gene was used as the internal control. Dissociation curves were performed at the end of amplification for validating data quality. Each individual sample was run in triplicate and the average critical threshold cycle (Ct) was used for calculating relative quantification by fold-change and statistical significance.

### Bioinformatics

An unreleased version of the High Throughput Gene Ontology Functional Annotation Toolkit (HTGOFAT, ) was utilized to assign updated Gene Ontology numbers [21], Enzyme Commission [56] numbers, mappings to Kyoto Encylopedia of Genes and Genomes (KEGG) Pathways [57] and updated definitions. Statistics related to over representation of functional categories were performed using a Fisher Exact statistic methodology similar to that described by Al-Shahrour *et al *[58]. In brief, differentially expressed genes (*P *< 0.001) were selected and separated based on regulation directions (up or down) in each comparison. Data mining to PubMed IDs was performed using a beta version module within HTGOFAT that searched PubMed abstracts using upon experimental conditions or terms (e.g. chicken and *Salmonella*) that co-occur with gene names and symbols that are represented within a given dataset. Additionally, differentially regulated genes were mapped to Protein Information Resource (PIR) keywords [59] and COG [60] functional annotations through the use of primary mappings with HTGOFAT and subsequent mapping and clustering using the Database for Annotation, Visualization and Integrated Discovery (DAVID) [61].

## Authors' contributions

HC carried out the microarray experiments, analyzed data and drafted the manuscript. CS was responsible for heterophil isolation, *in vitro *stimulation, RNA isolation, and revision of the manuscript. MK contributed to experiment design and data interpretation. SD contributed to functional annotation and analyses. XL assisted in data processing. IP provided genetic lines. HZ designed the microarray, provided the concepts of the study, and revised the manuscript. All authors submitted comments, read and approved the final manuscript.

## Supplementary Material

Additional file 1 **Differentially expressed genes in the comparison AI to AN**. The file contains all differentially expressed (*P *< 0.001 with fold-change > 1.5 or < -1.5) genes shown in the comparison between infected and non-infected cells within line A. Click here for file

Additional file 2 **Differentially expressed genes in the comparison BI to BN**. The file contains all differentially expressed (*P *< 0.001 with fold-change > 1.5 or < -1.5) genes shown in the comparison between infected and non-infected cells within line B. Click here for file

Additional file 3 **Differentially expressed genes in the comparison AN to BN**. The file contains all differentially expressed (*P *< 0.001 with fold-change > 1.5 or < -1.5) genes shown in the comparison of non-infected cells between different lines. Click here for file

Additional file 4 **Differentially expressed genes in the comparison AI to BI**. The file contains all differentially expressed (*P *< 0.001 with fold-change > 1.5 or < -1.5) genes shown in the comparison of infected cells between different lines. Click here for file

Additional file 5 **Primers used for qRT-PCR**. This file contains primers sequences and amplified PCR product sizes of genes chosen for qRT-PCR confirmation. Click here for file

Additional file 6 **Expression differences found with the microarray compared with the qRT-PCR**. This file contains expression differences found with the microarray compared with the qRT-PCR. Bold are differences in expression levels found with the microarray (*P *< 0.001) as well as the qRT-PCR (*P *< 0.05). Click here for file
