# Supplementary material for: Gene expression profiling in chicken heterophils with Salmonella enteritidis stimulation using a chicken 44 K Agilent microarray
Source: BMC Genomics. 2008 Nov 6;9:526. doi: 10.1186/1471-2164-9-526 (PMC2588606; doi:10.1186/1471-2164-9-526)
Supplement: Additional file 5 — Primers used for qRT-PCR. This file contains primers sequences and amplified PCR product sizes of genes chosen for qRT-PCR confirmation. [file 1471-2164-9-526-S5.doc]

Primers used for qRT-PCR

| Accession No. | Gene Name | Forward Sequence (5’-3’) | Reverse Sequence (5’-3’) | PCR Product Size (bp)* |
| --- | --- | --- | --- | --- |
| NM_205518 | β-actin | ACGTCTCACTGGATTTCGAGCAGG | TGCATCCTGTCAGCAATGCCAG | 298 |
| L34553 | CCL4 | TACTACGAGACCAACAGCCAGT | TGTCTCCACATCCATTCTATCC | 153 |
| AJ309540 | Interleukin 6 (IL6) | AGGACGAGATGTGCAAGAAGTTC | TTGGGCAGGTTGAGGTTGTT | 78 |
| AJ851659 | CD80 antigen | CAGAGTCTCCAGTCTTCACCAG | GGAAAACCTCCATGAGAAGAAC | 173 |
| Y14971 | CXC chemokine K60 (K60) | AGACTCATTCCAAGTTCATCCA | TTTGTTCTTTGCTTTAGGATGC | 216 |
| M64990 | Prostaglandin-endoperoxide synthase 2 (PTGS2) | TCGAGATCACACTTGATTGACA | TTTGTGCCTTGTGGGTCAG | 230 |
| AF176086 | Similar to NUMB protein (NUMB) | CAGCAGACGTTCCCTCAGTA | ATCACTTGAGAAGGGGTTGG | 213 |
| U20338 | Interferon regulatory factor 7 (IRF7) | ATCCCTTGGAAGCACAACGCC | CTGAGGCAACCGCGTAGACCTT | 223 |
| AF082329 | Caspase 6, apoptosis-related cysteine peptidase (CASP6) | CAGAGGAGACAAGTGCCAGA | CCAGGAGCCGTTTACAGTTT | 250 |

*All of primers are designed with melting temperature around 59 ºC.
