# Supplementary material for: Gene expression profiling in chicken heterophils with Salmonella enteritidis stimulation using a chicken 44 K Agilent microarray
Source: BMC Genomics. 2008 Nov 6;9:526. doi: 10.1186/1471-2164-9-526 (PMC2588606; doi:10.1186/1471-2164-9-526)
Supplement: Additional file 6 — Expression differences found with the microarray compared with the qRT-PCR. This file contains expression differences found with the microarray compared with the qRT-PCR. Bold are differences in expression levels found with the microarray (P < 0.001) as well as the qRT-PCR (P < 0.05). [file 1471-2164-9-526-S6.doc]

**Expression differences found with the microarray compared with the qRT-PCR**

| Gene Name | AI/AN fold-change^a^ | | BI/BN fold-change^a^ | | AI/BI fold-change^a^ | | AN/BN fold-change^a^ | |
| --- | --- | --- | --- | --- | --- | --- | --- | --- |
|  | Microarray | qRT-PCR | Microarray | qRT-PCR | Microarray | qRT-PCR | Microarray | qRT-PCR |
| Chemokine (C-C) ligand 4 (CCL4) | **7.30** | **3.76** | **3.59** | **1.73** | 2.48 | 2.09 | 1.22 | 1.04 |
| Interleukin 6 (IL6) | **11.39** | **6.59** | **8.96** | **5.02** | -1.11. | 1.01 | -1.41. | 1.29 |
| CD80 antigen | **3.85** | **4.18** | 1.53 | **2.31** | 1.60 | 1.25 | -1.57 | 1.45 |
| CXC chemokine K60 (K60) | **8.93** | **17.14** | **4.12** | **21.46** | 2.15 | 1.67 | -1.01 | 2.09 |
| Prostaglandin-endoperoxide synthase 2 (PTGS2) | **3.14** | **4.90** | **3.19** | **7.87** | 2.12 | **1.52** | 2.15 | **2.44** |
| Similar to NUMB protein (NUMB) | -1.08 | 1.07 | **-2.29** | **-2.51** | **2.18** | **1.87** | 1.02 | 1.15 |
| Interferon regulatory factor 7 (IRF7) | -1.16 | -1.31 | 1.02 | -1.59 | **2.03** | **1.59** | **2.40** | 1.31 |
| Caspase 6, apoptosis-related cysteine peptidase (CASP6) | 1.32 | 1.68 | 1.06 | 1.42 | -2.15 | -1.48 | **-2.68** | **-1.74** |

a,: Bold are differences in expression levels within the comparison found with the microarray (*P* < 0.001) as well as the qRT-PCR (*P* < 0.05).
